# Public health round-up

**DOI:** 10.2471/BLT.14.011214

**Published:** 2014-12-01

**WHO guidance for frontline workers**

A safe burial team prepares to inter a person believed to have died of Ebola virus disease in Freetown, Sierra Leone. Two new sets of WHO guidance – one on safe burial and the other on personal protective equipment (PPE) – are important resources to help stop the outbreak that is ravaging parts of western Africa.

http://www.who.int/csr/resources/publications/ebola/safe-burial-protocol

http://www.who.int/csr/resources/publications/ebola/ppe-guideline

**Figure:**
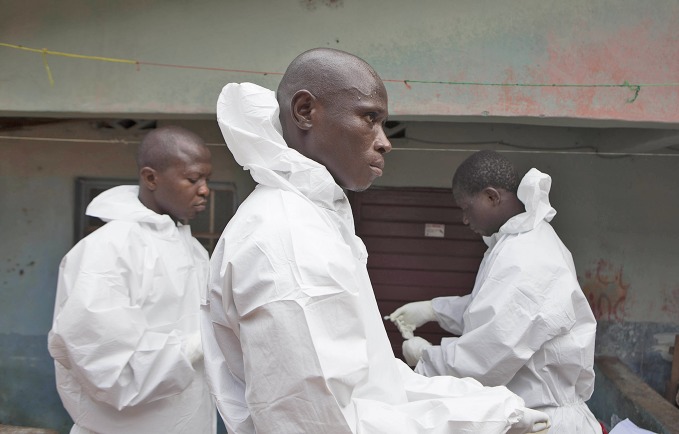
WHO/C Black

## Nutrition for 21st century

An ambitious new agenda – in the form of a Political Declaration and Framework for Action to improve nutritional health for the next 10 years – was presented to a major United Nations conference in Rome, Italy, last month.

The agenda addresses the nutritional problems of the 21st century, including undernutrition, which affects an estimated 805 million people, as well as micronutrient deficiencies, obesity or other consequences of poor diet, which affect a third of the world’s population.

The political declaration refers to six goals that were approved by the World Health Assembly in May: a 40% reduction in children less than 5 years of age who are stunted; a 50% reduction of anaemia in women of reproductive age; a 30% reduction in low birth weight; the prevention of a further increase in childhood overweight; a 50% increase in the rate of exclusive breastfeeding in the first 6 months; and a reduction in childhood wasting to less than 5%.

“If current trends continue, the world will not meet these goals in a decade,” said Dr Francesco Branca, director of the Department of Nutrition for Health and Development at the World Health Organization (WHO) in Geneva. “The number of stunted children aged under 5 years is projected to be 128 million in 2025, against a target of 100 million, and this is perhaps the one of the six areas where most progress is being made.”

“The six global nutrition targets are also being discussed in negotiations around the post-2015 development agenda,” Branca said. “If they are adopted, with 2030 as a time horizon, we expect to see nutrition dramatically improving within one generation.”

To guide countries working towards the new goals to improve nutritional health, WHO released a new set of policy briefs and contributed to the *Global nutrition report 2014: actions and accountability to accelerate the world’s progress on nutrition,* which was published last month by the International Food Policy Research Institute.

The conference from 19 to 21 November was organized by the Food and Agricultural Organization and WHO.

http://www.who.int/nutrition/topics/WHO_FAO_announce_ICN2

## Measles progress stalled

The number of deaths from measles – a vaccine-preventable disease – increased to an estimated 145 700 last year up from 122 000 in 2012, according to new data published in the WHO *Weekly Epidemiological Report* and the Centers for Disease Control and Prevention’s *Morbidity and Mortality Weekly Report* last month.

The increase was mainly because of large outbreaks the Democratic Republic of the Congo, Nigeria, Pakistan and Somalia and smaller outbreaks in other parts of the world.

Progress in vaccinating children is stalled in the WHO Eastern Mediterranean region, where weak health systems, conflict and population displacement have hampered vaccination work. The European region of WHO has also seen an upsurge in measles cases with outbreaks in several countries including Georgia, Turkey and Ukraine.

In 2010, the World Health Assembly (WHA 63.18) established three milestones to be achieved by 2015 on the road towards measles eradication.

One milestone is to increase to 90% the percent of children aged 1 year who have received the first of two recommended doses of measles-containing vaccine (MCV1), but this coverage has been stagnant at around 83% since 2009.

The other goals – to reduce and maintain annual measles incidence to fewer than 5 cases per million and to reduce measles deaths by more than 95% of the estimated level for the year 2000 – are also off track.

“Poor progress in increasing measles vaccination coverage has resulted in large outbreaks of this highly contagious disease, throwing the 2015 elimination targets off track,” said Dr Peter Strebel from the WHO Department of Immunization, Vaccines, and Biologicals.

http://www.who.int/wer

## Fewer overdose deaths

New WHO guidelines recommend that naloxone – a safe drug with a low risk of serious side-effects – be made available to people who are likely to witness an overdose of heroin or other opioids.

In most countries, naloxone is only accessible through hospitals and ambulance crews. The new guidelines recommend that it should be made available to the friends, relatives and partners of people who use drugs, as well as social workers.

Expanding access to this life-saving drug could reduce deaths from opioid overdose, estimated at 69 000 a year globally, according to research in the journal *Community management of opioid overdose* that was released last month.

http://www.who.int/substance_abuse/publications/management_opioid_overdose

**Cover photo**

A boy is tested for malaria in the city of Dajabón in the Dominican Republic. Malaria cases declined by 39% between 2012 and 2013 in the Dominican Republic, one of 21 countries in WHO’s Region of the Americas where the disease is still endemic.

**Figure:**
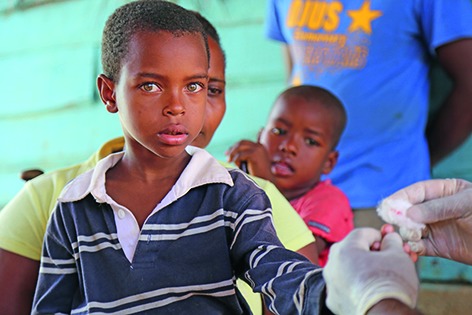
WHO/PAHO/S Oliel

## Cleaner indoor air

WHO released new guidelines last month recommending measures that countries can take to improve the quality of air inside people’s homes and thus prevent millions of deaths associated with household air pollution.

According to the WHO *Guidelines for indoor air quality: household fuel combustion*, countries where people burn fuels such as unprocessed coal and kerosene inside their homes should introduce the use of cleaner heating and cooking technologies, such as liquefied petroleum gas, biogas, natural gas, ethanol and electricity.

The guidelines also propose targets for reducing emissions of health-damaging pollutants from domestic cook-stoves, space heaters and fuel-based lamps.

“Ensuring cleaner air in and around the home is fundamental to reducing the burden of disease from air pollution, especially in low- and middle-income countries,” said Dr Maria Neira, director for Public Health, Environmental and Social Determinants of Health at WHO

The guidelines follow the release of data in March showing that an estimated 4.3 million people die every year from diseases associated with household air pollution, including stroke, ischaemic heart disease, chronic obstructive pulmonary disease and childhood pneumonia.

Nearly 3 billion people worldwide still lack access to clean fuels and technologies for cooking, heating and lighting.

http://www.who.int/indoorair/guidelines/hhfc

## Drug-resistant TB crisis

Globally an estimated 480 000 people developed multidrug-resistant tuberculosis (MDR–TB) in 2013 and 210 000 people died of this form of the disease, as severe epidemics continued to rage in some regions, particularly eastern Europe and Central Asia, according to the *Global tuberculosis report 2014*, released in October.

Treating a patient for MDR–TB – defined as resistance to isoniazid and rifampicin – takes up to two years and is expensive, while cure rates are below 50% in most countries.

Since 2009, laboratories in more than 100 countries have been using new rapid diagnostic tests for MDR–TB. As a result, the number of people diagnosed with this form of tuberculosis has tripled globally, reaching 136 000 in 2013, but only 97 000 of them were put on treatment in that year, according to the WHO report.

“With improved diagnostic tools and access [to them] we are now detecting and treating more cases,” says Dr Karin Weyer, coordinator for Laboratories, Diagnostics and Drug Resistance at WHO. “But the gap between detecting and actually getting people started on treatment is widening and we urgently need increased commitment and funding to test and successfully treat every case.”

A special supplement to this year’s WHO report marks 20 years of anti-tuberculosis drug-resistance surveillance. It also describes the response to the MDR–TB crisis to date and proposes priority actions that should be taken, from prevention to cure.

http://www.who.int/tb/publications/global_report

## New WHO leader in Africa

Dr Matshidiso Moeti was elected the new regional director of the WHO African Region at its Regional Committee meeting of the 47 ministers last month in Cotonou, Benin.

She is the first woman and first citizen of Botswana to be nominated to the post and previously served as deputy regional director at the WHO Regional Office for Africa. Pending confirmation of her nomination by WHO's Executive Board next month, she is due to take up her duties on 1 February when the 10-year term of Dr Luis Sambo as regional director ends.

Moeti qualified in medicine at the Royal Free Hospital School of Medicine in London in 1978 and public health at the London School of Hygiene and Tropical Medicine in 1986. She has over 35 years of public health experience, including at WHO since 1999 as well as the United Nations Children’s Fund, UNAIDS and the Botswana Ministry of Health.

http://www.afro.who.int/en/sixty-fourth-session/rd-nomination/biography-dr-moeti.html

**Looking ahead**

**26 January–3 February – WHO Executive Board meeting in Geneva, Switzerland**
http://apps.who.int/gb/e/e_eb136.html
**26–31 January – Prince Mahidol Award Conference, Bangkok, Thailand**
http://www.pmaconference.mahidol.ac.th

